# Formulating InVO_4_/α-Fe_2_O_3_ Heterojunction Composites for Photocatalytic Tetracycline Hydrochloride Degradation

**DOI:** 10.3390/nano14171441

**Published:** 2024-09-04

**Authors:** Haoxu Chang, Yayang Wang, Panzhe Qiao, Bo Sun, Zhengbang Wang, Fei Song

**Affiliations:** 1 Manchester Metropolitan Joint Institute, Hubei University, Wuhan 430062, China; 202131123001009@stu.hubu.edu.cn; 2 School of Materials Science and Engineering, Hubei University, Wuhan 430062, China; 202311113010161@stu.hubu.edu.cn; 3 Shanghai Advanced Research Institute, Chinese Academy of Sciences, Shanghai 202104, China; qiaopz@sari.ac.cn (P.Q.); sunb@sari.ac.cn (B.S.)

**Keywords:** tetracycline hydrochloride, InVO_4_/α-Fe_2_O_3_, heterojunction, photocatalysis

## Abstract

This study reports the synthesis of InVO_4_/α-Fe_2_O_3_ heterojunction photocatalysts with different stoichiometric ratios via a two-step hydrothermal synthesis reaction. The prepared photocatalysts were characterized by XRD, SEM, TEM, XPS, and other methods. The prepared composites exhibited good photocatalysis of tetracycline hydrochloride. Among the InVO_4_/α-Fe_2_O_3_ heterojunction photocatalysts with different ratios, the InVO_4_/0.25α-Fe_2_O_3_ photocatalyst showed the highest degradation rate for 20 mg L^−1^ tetracycline hydrochloride. After three photocatalytic runs, it still exhibited excellent stability and reusability. Meanwhile, this study also found that superoxide radical anion (-O^2−^), electron (e^−^), hydroxyl radical (·OH), and photogenerated hole (h^+^) are the basic active substances in the photocatalytic process.

## 1. Introduction

Over the past few years, the detection of pharmaceuticals, notably antibiotics, in water sources has risen because of their extensive use in human and agricultural sectors [1]. Despite their importance for health and safety, their overuse threatens ecosystems and human health [2,3,4,5,6]. Tetracycline hydrochloride (TC) is among the most frequently used antibiotics worldwide. It is widely used in human and animal infection treatment and other medical aspects, offering the benefits of broad-spectrum antibacterial activity at a moderate cost [7,8]. However, its lengthy half-life and poor metabolic efficiency pose a significant and urgent environmental threat. Therefore, from the perspective of a healthy human life and socially sustainable development, it is crucial to develop technology that will help achieve environmentally friendly, green, and clean production to degrade TC [9,10].

Compared with traditional technologies of removing contaminants, such as flocculation, membrane separation, coagulation, and biodegradation, photocatalysis technology has attracted attention because of its high efficiency, recyclability, and economic advantages [11,12,13]. Photocatalysis is a type of chemical reaction that uses a unique material called a semiconductor photocatalyst and visible light to break down harmful substances. Using this technology to create potent oxidation free radicals, oxidize, and eliminate contaminants is the basic idea behind photocatalytic oxidation technology. The use of photocatalysis techniques, which combine solar technology and heterogeneous catalysis, has made it possible to remove antibiotics from wastewater economically and sustainably [14]. The use of semiconductor-based photocatalysis technology as an environmentally friendly means of eliminating contaminants from the environment has grown during the last several decades [15,16,17].

Even though semiconductor photocatalysis technology has advanced quickly, the low separation efficiency of photogenerated electron–hole pairs, restricted mass transfer, weak visible light absorption, and catalyst inactivation caused by pollutants and their byproducts make it difficult for current photocatalysis technology to meet industrial-scale needs [16]. Therefore, achieving the successful utilization of solar energy and large-scale deployment of TC removal from wastewater using ecological means requires choosing a suitable catalyst that is essential for photocatalytic degradation [17,18]. Currently, scientists are concentrating on using active materials in visible light. Narrow-band gap oxides are better suited for photocatalysis because they have a broader absorption spectrum in visible light, such as BiVO_4_ [19,20], TiO_2_ [21], CdS [22], and g-C_3_N_4_ [23], which have been widely studied for wastewater treatment under visible light. Indium vanadate (InVO_4_), a narrow bandgap substance, is a successful photocatalyst due to its unique properties. These include beneficial optical and electronic characteristics, biocompatibility, and broad visible light absorption. Notably, it is non-toxic and demonstrates outstanding resistance to chemicals and photo corrosion [18,19,20,21,22,23].

However, single-component photocatalysts typically exhibit lower light absorption and higher electron–hole pair complexation rates [24]. This is also true for the one-component InVO_4_ catalyst material, which does not have a high enough photocatalytic efficiency in visible light because of the rapid rate of electron–hole pair complexation generated in the narrow band gap [25]. In fact, many scientists have recently adopted heterogeneous composite materials to treat wastewater. Ivanov et al. prepared Fe_3_O_4_ and Zn-Al-LDH composite adsorbents with high exchange capacity and selectivity by the precipitation method, and the maximum adsorption and exchange capacity for U(VI) reached 268.65 ± 22.22 mg g^−1^, which is very suitable as adsorbents for U(VI) removal [26]. Balybina et al. investigated a series of adsorbent materials based on layered double hydroxide (LDH). They found that Co-Fe LDH showed the highest adsorption capacity in seawater, which is important for the nuclear energy industry and environmental protection [27]. Furthermore, it has been demonstrated that doping and heterojunction construction can enhance the photoelectrochemical performance of a photocatalyst [18,20,28,29]. Zhang et al. investigated Bi-Au/SiO_2_ tandem bimetallic catalysts, which were monitored using near-environmental pressure X-ray photoelectron spectroscopy and were of great significance for an in-depth understanding of the catalytic mechanism and optimization of catalyst design [30]. Performance under visible light illumination degradation can be enhanced by doping certain metals (i.e., Fe, Ag, and Ce). In order to enhance photocatalytic activity, several researchers and scholars have compounded InVO_4_ with other materials. For example, InVO_4_/CeVO_4_ empty nanoribbons with non-homogeneous interfaces and internal cavities and a TOC removal rate of 90.4% TC after 135 min of radiation were synthesized by Ding et al., which provided a new strategy for the design of high-efficiency photocatalysts [28]. Tamtam et al. successfully synthesized ZnWO_4_ nanorod-modified InVO_4_ nanosheets, which were used for the highly efficient decomposition of tetracycline (TC) with an enhanced performance of 7.6 times, demonstrating their potential in the photocatalytic field [25].

Previous studies have shown that a heterojunction coupled with α-Fe_2_O_3_ has a low photogenerated carrier complexation rate and a strong oxidation capability and can directly burst reducing electrons and oxidizing holes simultaneously by charge transfer at low potentials [31,32,33]. As an n-type narrow semiconductor, α-Fe_2_O_3_ has an excellent optical bandgap (1.9–2.2 eV) and can absorb light in the visible region [34,35,36]. Wang et al. prepared and obtained α-Fe_2_O_3_ using a straightforward hydrothermal method. Additionally, α-Fe_2_O_3_ is an excellent option for photocatalysts in wastewater treatment because of its low cost, good chemical stability, high abundance, high visible light utilization efficiency, non-toxicity, and environmental friendliness [37,38,39,40]. The rGO/InVO_4_/Fe_2_O_3_ Z-scheme heterostructured photocatalysts developed by Kumar et al. showed excellent results in CO_2_ photoreduction to methanol, demonstrating the potential of InVO_4_/Fe_2_O_3_ heterostructures in photocatalysts [41]. Consequently, the combination of InVO_4_ and α-Fe_2_O_3_ can overcome the limitations of the two materials and further enhance the utilization of sunlight by improving the photoelectrochemical properties of the photocatalyst. The aim of this paper is to gain insight into the structural and optical properties of heterojunction materials to improve the photocatalytic activity of InVO_4_ materials and to show the effect of InVO_4_/α-Fe_2_O_3_ materials in degrading TC.

**Figure 1:**
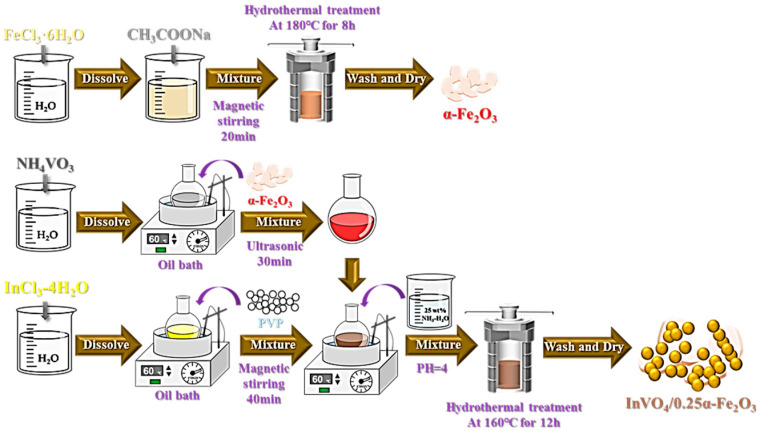
Schematic diagram of the synthesis steps of InVO_4_/α-Fe_2_O_3_.

In this study, InVO_4_ composite materials doped with varying ratios of α-Fe_2_O_3_ were prepared via the hydrothermal method, as shown in Figure 1 and the Experimental Methods Section. The impact of α-Fe_2_O_3_ doping on the properties of InVO_4_, including structure, morphology, and photoelectrochemical properties, was investigated. The visible photocatalytic activity of the prepared α-Fe_2_O_3_-doped InVO_4_ composites was evaluated by intensively investigating the photocatalytic degradation of a tetracycline hydrochloride solution, for example, by further studying the degradation properties of the materials by varying the conditions of catalyst addition and initial TC concentration, respectively.

## 2. Experimental Methods

### 2.1. Material Synthesis

#### 2.1.1. Synthesis of α-Fe_2_O_3_

A hydrothermal method was used to synthesize α-Fe_2_O_3_. First, 0.540 g (0.2 mmol) of FeCl_3_·6H_2_O was dissolved in 22 mL of a solvent mixture of deionized water and ethanol in a volume ratio of 1:10. Then, 1.6 g of CH_3_COONa was added and stirred for 20 min. Then, the mixture was transferred to a 50 mL Teflon-lined stainless autoclave and heated at 180 °C for 8 h in a blast drying oven. After cooling, the solids were washed thrice with ultrapure water and anhydrous ethanol by high-speed centrifugation. The precipitate was dried in a vacuum drying oven at 60 °C for 18 h to obtain α-Fe_2_O_3_.

#### 2.1.2. Synthesis of InVO_4_/α-Fe_2_O_3_ Composites and InVO_4_

InVO_4_/α-Fe_2_O_3_ composites with different ratios were synthesized by a hydrothermal method. Firstly, 0.293 g (0.1 mmol) of InCl_3_·4H_2_O and 0.117 g (0.2 mmol) of NH_4_VO_3_ were dissolved in two beakers with deionized water and heated to 60 °C. Then, different amounts of α-Fe_2_O_3_ particles were added proportionally to the NH_4_VO_3_ solution, stirred, and ultrasonicated for 30 min. Next, the mixed solution was added to the InCl_3_·4H_2_O solution with 0.2 g of PVP and was stirred continuously for 40 min, and then, its pH was adjusted to about 4 with 25 wt% NH_3_-H_2_O. Then, the mixture was transferred to a 100 mL Teflon-lined stainless autoclave and heated at 160 °C for 12 h in a blast drying oven. After cooling, the solids were washed thrice with ultrapure water and anhydrous ethanol by high-speed centrifugation. The precipitate was dried in a vacuum drying oven at 60 °C for 18 h to obtain α-Fe_2_O_3_/InVO_4_ composites. Using this process, InVO_4_/α-Fe_2_O_3_ composites were produced as InVO_4_/0.25α-Fe_2_O_3_ InVO_4_/0.5α-Fe_2_O_3_, InVO_4_/0.75α-Fe_2_O_3_, and so on, with varying molar ratios of α-Fe_2_O_3_ to InVO_4_. By the same synthesis procedure, pure InVO_4_ photocatalysts were synthesized for control studies without the addition of α-Fe_2_O_3_ particles. The synthetic route is shown in Figure 1.

### 2.2. Characterization

The size and morphology of the catalysts were studied by scanning electron microscopy (SEM, ZEISS Gemini 300, Jena, Germany) and EDS elemental analysis. The sample’s crystal structure was analyzed by an X-ray diffractometer (XRD) at beamline BL14B1, Shanghai Synchrotron Radiation Facility (SSRF), Shanghai, China. The wavelength used in the XRD analysis was 0.6888 Å (18 keV X-ray energy). The beam size was about 400 μm × 400 μm. The detector used to collect data was Mythen 1K (Dectris Inc., Baden, Switzerland). Al-Kα monochromatic X-ray photoelectron spectroscopy (XPS) was used to calibrate C 1s peak energy (equal to 284.8 eV). Ultraviolet–visible diffuse reflectance spectroscopy (UV-vis) was conducted with an ultraviolet–visible spectrophotometer (Shanghai Jinghua, Shanghai, China, UV-1800).

### 2.3. Photocatalytic Experiments

The visible light irradiation-degraded TC served as a proxy for the materials’ photocatalytic activity. The light source was a 300 W xenon lamp (HSX-F300, NBeT, Patna, India) with a 400 nm cut-off filter. For every photocatalysis experiment, 50 mg of photocatalyst particles and 100 mL of TC solution (20 mg L^−1^) were introduced to a quartz reactor. Next, the reactor was stirred in the dark for 40 min to equilibrate the adsorption and desorption reactions to construct the catalyst. Subsequently, the xenon lamp light source was switched on, and magnetic stirring was timed for ninety minutes. During the xenon lamp irradiation, 3 mL of water samples were taken at 15 min intervals, and the solid catalyst particles were removed via centrifugation. Using a UV–visible spectrophotometer (UV-1800, Shanghai Jinghua), the TC in the filtrate was examined. The photocatalytic degradation efficiency calculation formula is as follows:

% Photocatalytic degradation = [(C_0_ − C_t_)/C_0_] × 100 (1)

The initial concentration of TC, designated as C_0_, was contrasted with the instantaneous concentration, designated as C, following the sampling analysis.

## 3. Results and Discussion

### 3.1. The Wavelength Used in XRD Analysis at the Synchrotron Beamline

**Figure 2:**
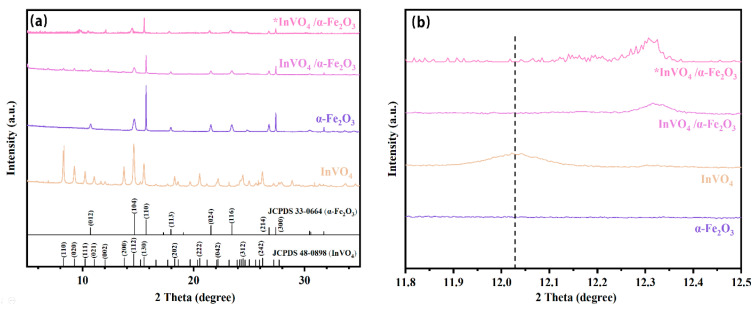
XRD diffraction patterns of InVO_4_, α-Fe_2_O_3_, InVO_4_/0.25α-Fe_2_O_3_, and *InVO_4_/0.25α-Fe_2_O_3_ (InVO_4_/0.25α-Fe_2_O_3_ after degradation once) in the range of (**a**) 2θ = 5–35° and (**b**) 2θ = 11.8–12.5°.

Figure 2a displays the XRD spectra of InVO_4_, α-Fe_2_O_3_, InVO_4_/0.25α-Fe_2_O_3_, and *InVO_4_/0.25α-Fe_2_O_3_ (InVO_4_/α-Fe_2_O_3_ after degradation once). The InVO_4_/α-Fe_2_O_3_ composites exhibit diffraction peaks at 8.2°, 9.2°, 10.2°, 11.1°, 12.0°, and 15.54°, which are comparable to the pristine InVO_4_ and α-Fe_2_O_3_ and correlate to an orthorhombic crystalline structure with an orthorhombic InVO_4_ plane (110), (020), (111), (021), (002), and (130) (JCPDS 48-0898). The displacement of (002) planar InVO_4_ at roughly 12° is displayed in Figure 2b. The hematite (α-Fe_2_O_3_) planes (012), (104), (110), (113), (024), (116), (214), and (300) correspond to values of approximately 10.7°, 14.7°, 15.7°, 17.9°, 21.5°, 23.5°, 26.8°, and 27.4° at 2θ (JCPDS 33-0664). The above results indicate the successful synthesis of the InVO_4_/α-Fe_2_O_3_ complex photocatalysts.

### 3.2. Surface Area and Pore Structure

**Figure 3:**
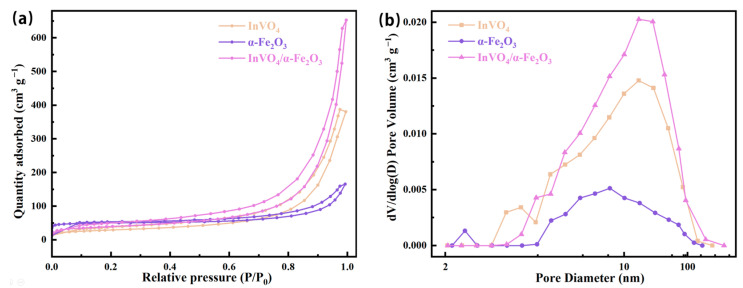
(**a**) N_2_ adsorption–desorption isotherm and (**b**) the pore size distribution curves of the samples.

The BET-specific surface area and the corresponding pore size distribution of the synthesized samples were analyzed by N_2_ adsorption–desorption isotherms, as shown in Figure 3. According to the IUPAC classification, the N_2_ adsorption–desorption isotherms of all the synthesized samples can be categorized as type IV adsorption isotherms, indicating that the samples are mesoporous [41]. The pore size distribution curves calculated by the BJH method further reveal that the samples have mesoporous structures, and the average pore sizes of InVO_4_/0.25α-Fe_2_O_3_, InVO_4_, and α-Fe_2_O_3_ are 27.1070 nm, 21.7435 nm, and 22.7819 nm, respectively, with a more centralized distribution of particle sizes, as shown in Figure 3b. A comparison of the specific surface area of these samples shows that the specific surface area of InVO_4_/0.25α-Fe_2_O_3_ 139.1711 m^2^ g^−1^ is greater than that of InVO_4_ (103.1375 m^2^ g^−1^) in the presence of α-Fe_2_O_3_ doping. According to the data, it can be seen that the surface area of the catalyst was prompted to be enlarged after the complexation of InVO_4_ with α-Fe_2_O_3_. In the photocatalytic process, the good specific surface area and internal pore structure facilitated the multiple reflections and refractions of light, which improved the utilization efficiency of light, thus effectively increasing the photocatalytic activity [42,43].

### 3.3. Morphologic Structure Analysis

**Figure 4:**
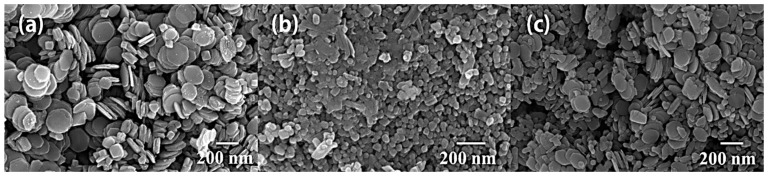
SEM images of (**a**) α-Fe_2_O_3_, (**b**) InVO_4_, and (**c**) InVO_4_/0.25α-Fe_2_O_3_ complex samples.

**Figure 5:**
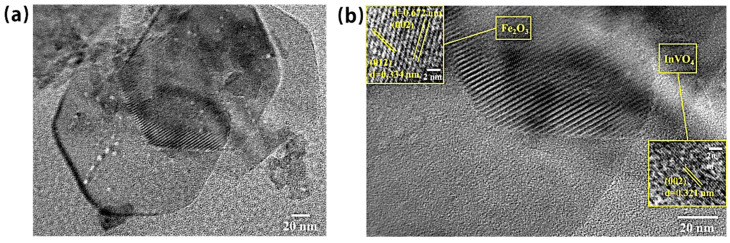
(**a**) TEM and (**b**) HRTEM images of InVO_4_/0.25α-Fe_2_O_3_ complex samples.

The SEM images of α-Fe_2_O_3_ in Figure 4a reveal that α-Fe_2_O_3_ has an overall regular shape, micro agglomerates, and a hexagonal flake-like shape. In contrast, InVO_4_ has a one-dimensional nanorod-like morphology or irregular microsphere-like agglomerates with nanometer sizes in Figure 4b. Figure 4c demonstrates that the InVO_4_ microspheres are attached to the hexagonal structure of α-Fe_2_O_3_, and InVO_4_ is well combined with α-Fe_2_O_3_. The transmission electron micrograph (TEM) of the InVO_4_/0.25α-Fe_2_O_3_ material is displayed in Figure 5. InVO_4_ point-like crystals are present on the α-Fe_2_O_3_ nanosheets, as shown in Figure 5a. There are two distinct crystal plane spacings in α-Fe_2_O_3_ (Figure 5b), which can be identified from the high-magnification transmission electron microscope image. These can be compared to the standard card and shown to correspond to the (012) and (002) crystal planes of α-Fe_2_O_3_, respectively, and the (002) crystal plane of InVO_4_ with the 0.321 nm lattice spacing.

### 3.4. Composition and Valence State Analysis

**Figure 6:**
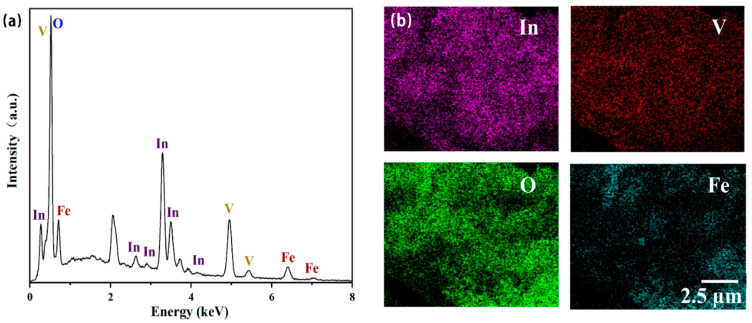
(**a**) EDS elemental mapping of InVO_4_/0.25α-Fe_2_O_3_ complexes and (**b**) the corresponding elemental mapping for In, V, O and Fe.

The EDS elemental mapping in Figure 6 further verified the structure of the InVO_4_/0.25α-Fe_2_O_3_ heterojunction. The elemental maps of In, V, O, and Fe were identified from photographs of certain hues (Figure 6b), and these elemental maps demonstrated the presence of the InVO_4_/α-Fe_2_O_3_ heterojunction. The results showed that In accounted for 67.49% and Fe accounted for 32.96%; α-Fe_2_O_3_ was distributed in InVO_4_, forming an InVO_4_/0.25α-Fe_2_O_3_ catalyst.

**Figure 7:**
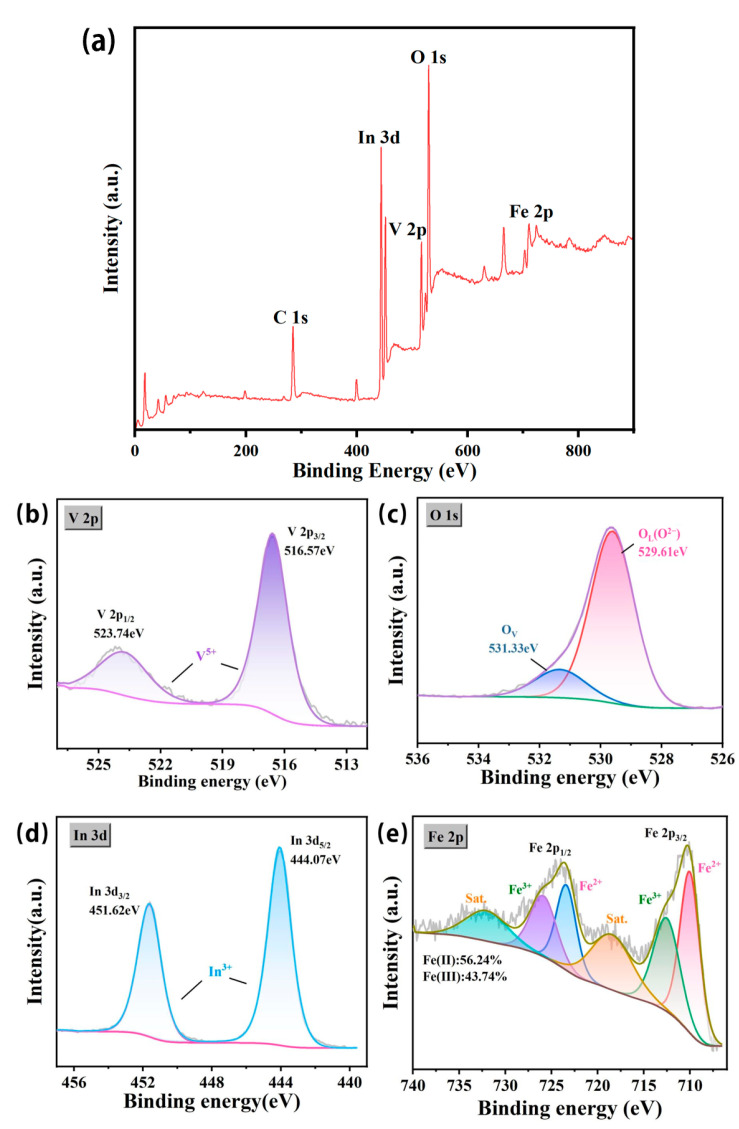
High-resolution XPS spectra of InVO_4_/0.25α-Fe_2_O_3_ complexes. (**a**) XPS survey spectra, (**b**) V 2p, (**c**) O 1s, (**d**) In 3d, and (**e**) Fe 2p.

X-ray photoelectron spectroscopy (XPS) was applied to examine the chemical composition and coordination environment of the InVO_4_/0.25α-Fe_2_O_3_ catalyst. As can be seen, the measured spectrum of InVO_4_/0.25α-Fe_2_O_3_ shows signals for the In, V, O, and Fe elements (Figure 7). The integration of 0.25α-Fe_2_O_3_ into InVO_4_ was confirmed by two peaks centered at 711 eV and 724.3 eV and two satellite peaks at 710.9 eV and 724.6 eV in the high-resolution spectra of Fe 2p (Figure 7e). Among them, Fe^2+^ and Fe^3+^ accounted for 56.24% and 43.74%, respectively. To uncover the impact of 0.25α-Fe_2_O_3_ doping on InVO_4_, additional analysis was conducted on the high-resolution spectra of V 2p, O 1s, and In 3d. The peaks at 444.07 eV and 451.62 eV for In 3d (Figure 7d) are ascribed to In 3d^5/2^ and In 3d^2/3^, respectively. The proportions were 38.68% and 61.32%, respectively. Lattice oxygen (O_L_) and chemisorbed oxygen (O_V_) (Figure 7c) are responsible for the two O 1s deconvolution peaks at 529.61 eV and 531.33 eV, respectively. The O_L_ peak of the InVO_4_/0.25α-Fe_2_O_3_ catalyst exhibits a comparable positive shift behavior to Fe-InVO_4_ synthesized by predecessors. The increase in oxygen vacancy is caused by the injection of α-Fe_2_O_3_ [20]. The typical V^5+^ peaks for V 2p (Figure 7b) are located at 516.57 eV and 523.74 eV, suggesting that V^5+^ ions have the potential to pick up electrons from adjacent oxygen vacancies.

### 3.5. Optical Property Analysis

**Figure 8:**
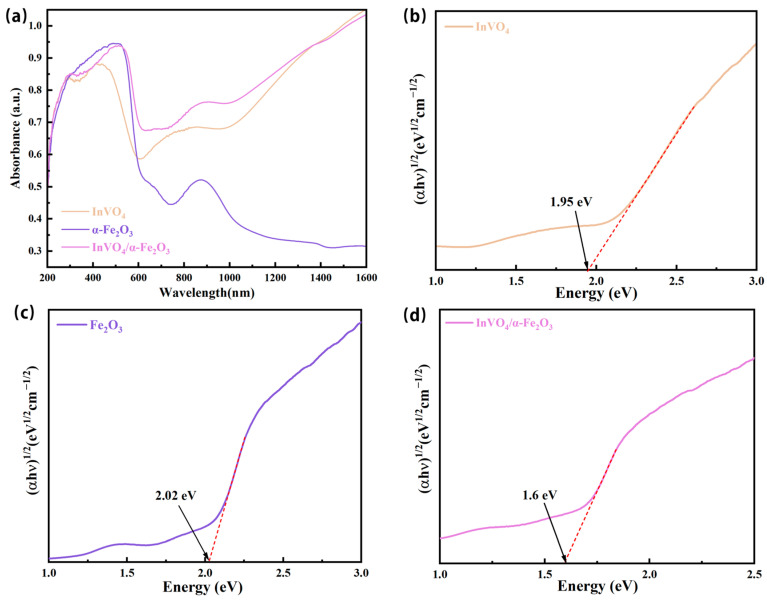
(**a**) Ultraviolet–visible absorption spectra of InVO_4_, α-Fe_2_O_3_, and InVO_4_/0.25α-Fe_2_O_3_ and the tauc plot for the band gap determination of (**b**) InVO_4_, (**c**) α-Fe_2_O_3_, and (**d**) InVO_4_/0.25α-Fe_2_O_3_.

To obtain a more profound comprehension of the impact of the coupling between InVO_4_ and α-Fe_2_O_3_ on the photocatalytic activity, UV-Vis DRS was utilized to analyze the optical characteristics of the photocatalysts. Figure 8a displays the UV-vis absorption spectra of InVO_4_, α-Fe_2_O_3_, and InVO_4_/0.25α-Fe_2_O_3_. The material with InVO_4_/0.25α-Fe_2_O_3_ has a strong absorption capacity in both the visible and infrared spectrums, with a UV spectral region of 300~400 nm and a visible spectral region of 400~600 nm.

Furthermore, as seen in Figure 8b–d, the band gaps of InVO_4_, α-Fe_2_O_3_, and InVO_4_/0.25α-Fe_2_O_3_ were computed using the Kubelka–Munk technique. These band gaps were determined to be 1.95 eV, 2.02 eV, and 1.6 eV, respectively [29]. It is simple to determine that the bandgap of the successful InVO_4_ and α-Fe_2_O_3_ composite narrows, improving the composites’ photoresponse to visible light irradiation.

### 3.6. Photocatalytic Degradation Performance

**Figure 9:**
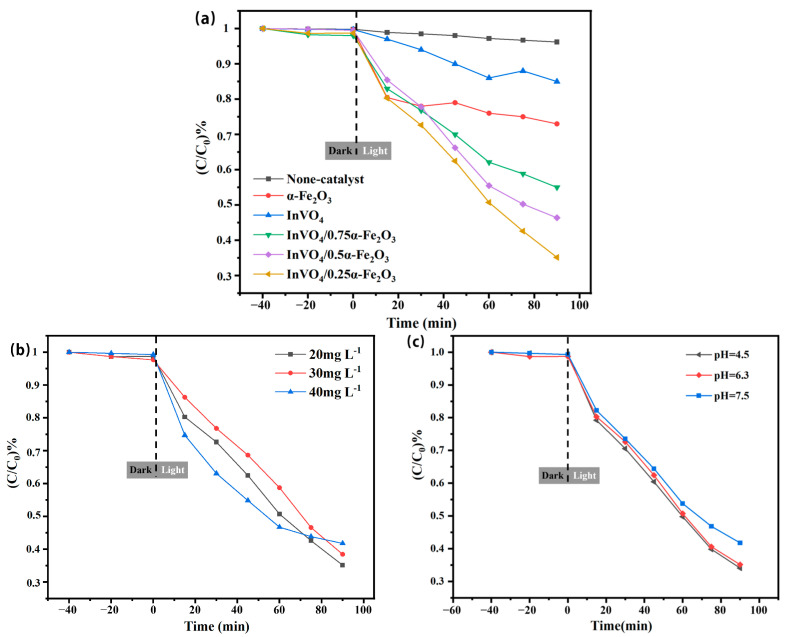
(**a**) The effect of irradiation time on the degradation efficiency of different photocatalysts (InVO_4_, α-Fe_2_O_3_, and InVO_4_/α-Fe_2_O_3_). (**b**) The effect of irradiation time on the degradation efficiency of different TC initial concentrations. (**c**) Initial pH effect of experimental conditions on the degradation of TC.

As illustrated in Figure 9, the photocatalytic activity of the material was evaluated by the degradation of TC under visible light at room temperature under dark adsorption for 40 min and visible light irradiation for 90 min. Control experiments were conducted at a catalyst dosage of 0 mg, and negligible fluctuations in TC concentration were observed. The dark adsorption efficiency of all samples was less than 5%. Furthermore, the photodegradation efficiency of the InVO_4_/0.25α-Fe_2_O_3_ composite was significantly enhanced compared with that of pure InVO_4_ or pure α-Fe_2_O_3_. The InVO_4_/0.25α-Fe_2_O_3_ catalyst exhibited the highest photocatalytic activity among the samples. The photocatalytic activity of the InVO_4_/0.5α-Fe_2_O_3_ and InVO_4_/0.75α-Fe_2_O_3_ composites decreased sequentially as the Fe content increased. Opportune lattice defects can be produced by doping α-Fe_2_O_3_ into InVO_4_, providing enough holes for the adsorption of TC and trapping photogenerated electrons. In addition, the heterojunction structure that forms at the junction interface facilitates the efficient migration and separation of the photogenerated electron–hole pair.

The degradation rate of TC showed a negative connection with the initial TC content (20 mg L^−1^ to 40 mg L^−1^) (Figure 9b). TC with an initial concentration of 40 mg L^−1^ degraded more quickly, presumably because of the catalyst’s increased propensity to bind TC in solution. On the other hand, the TC concentration dropped over time, and its ultimate degradation rate was the lowest. This is partially because the catalytic effectiveness decreased when the by-products of TC breakdown at 40 mg L^−1^ were more adhered to the catalyst surface than those at 20 mg L^−1^. Since tetracycline hydrochloride is an amphoteric antibiotic, another primary parameter influencing photocatalytic efficiency is the pH of the tetracycline solution. The photocatalytic breakdown of the catalyst at varying pH values is depicted in (Figure 9c). The figure shows that the catalyst’s rate of tetracycline hydrochloride degradation is greatest at a pH of 6.3, and in the solution at a pH of 4.5, the rate shows little variation. At pH 7.5, the rate of deterioration is reduced. Because TC is transformed into an anion or cation and electrostatic repulsion is created in front of the catalyst, the degrading efficiency of the catalyst may be diminished at high or low pH values [44].

### 3.7. Stability and Recyclability Properties

**Figure 10:**
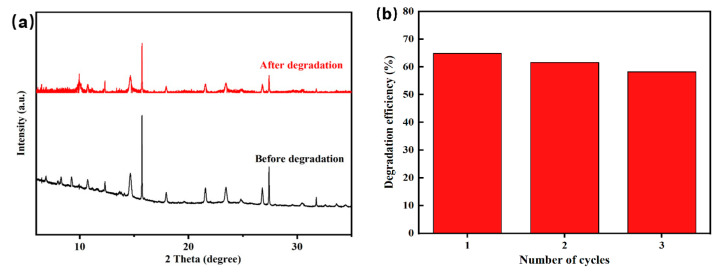
(**a**) XRD patterns of the InVO_4_/0.25α-Fe_2_O_3_ photocatalyst before and after the degradation of TC. (**b**) Reusability of the InVO_4_/0.25α-Fe_2_O_3_ photocatalyst.

Three cycles of TC degradation experiments were conducted (Figure 10a), which aimed to examine the stability and recoverability of the InVO_4_/0.25α-Fe_2_O_3_ photocatalyst. The outcomes show that the product’s absorption into the catalyst’s active sites may cause the modest drop in photocatalytic activity seen in the three cycles (Figure 10a). Nonetheless, the photocatalyst’s ability to catalyze remains potent. The XRD diffractograms of InVO_4_/0.25α-Fe_2_O_3_ before and after recycling are shown in Figure 10b. The findings of the XRD examination show that during the photocatalytic process, the phase composition of the InVO_4_/0.25α-Fe_2_O_3_ photocatalyst stays virtually unchanged, which suggests its outstanding stability after three cycles.

### 3.8. Photocatalytic Pollution Removal Mechanism

**Figure 11:**
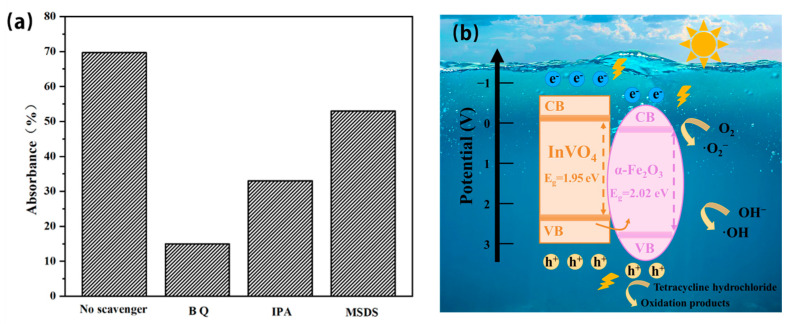
(**a**) Trapping experiments of the active species. (**b**) Possible mechanism of TC degradation by the InVO_4_/0.25α-Fe_2_O_3_ composite photocatalyst.

Reactive oxygen capture studies were conducted using various trapping agents to investigate the impact of distinct reactive groups on the photocatalytic mechanism (Figure 11a). Every trap prevented TC from degrading. Benzoquinone was found to have a notable impact on TC degradation, suggesting that superoxide radical oxidation is the primary mechanism involved in TC degradation, with the oxidation of hole and hydroxyl radicals also playing a role. As previously noted, a suggested mechanism for the degradation of TC was formulated based on the findings of the studies. It is suggested that TC is adsorbed both inside the internal spaces and on the catalyst’s surface at first. The catalyst’s variation in band gap makes it easier for photogenerated hole–electron pairs to form when exposed to visible light. Part of the O_2_ in solution is reduced to O_2_^−^ by the electrons in the conduction band, and part of the OH- in solution is oxidized to ·OH by the holes in the valence band. TC degrades gradually because of its reactions with reactive groups such as h*, O_2_, OH-, and so on [25,45,46]. The photo-induced electrons of the InVO_4_/0.25α-Fe_2_O_3_ catalyst are shown to flow between VB and CB in Figure 11b. During this electron transfer, a superoxide radical anion (·O_2_^−^) is formed because of the reduction of oxygen molecules. By contrast, when water molecules or hydroxyl ions are oxidized in the presence of Valance band gaps, hydroxyl radicals (·OH) are created. Tetracycline is broken down into oxidation products by these superoxide anions (·O_2_^−^) and hydroxyl radicals (·OH).

## 4. Conclusions

Using a two-step hydrothermal synthesis process, InVO_4_/α-Fe_2_O_3_ heterojunctions were successfully prepared with varying ratios of α-Fe_2_O_3_ to InVO_4_, and their ability to enhance the photocatalytic degradation of TC under visible light irradiation was examined. The InVO_4_/α-Fe_2_O_3_ composites have a higher degradation rate when compared with that of the single material, α-Fe_2_O_3_ or InVO_4_, alone. InVO_4_/0.25α-Fe_2_O_3_ is the optimized photocatalyst with the highest photocatalytic activity. In conclusion, there is exciting future research potential for the green photocatalytic material, the InVO_4_/α-Fe_2_O_3_ heterojunction photocatalysts.

## Data Availability

Data are contained within the article.
